# Mitochondrial network remodeling of the diabetic heart: implications to ischemia related cardiac dysfunction

**DOI:** 10.1186/s12933-024-02357-1

**Published:** 2024-07-18

**Authors:** Michael W. Rudokas, Margaret McKay, Zeren Toksoy, Julia N. Eisen, Markus Bögner, Lawrence H. Young, Fadi G. Akar

**Affiliations:** 1 Department of Internal Medicine, Section of Cardiovascular Medicine, Yale School of Medicine, New Haven, CT USA; 2 Department of Biomedical Engineering, Yale University Schools of Engineering and Applied Sciences, New Haven, CT USA; 3 Department of Biomedical Engineering, Electro-biology and Arrhythmia Therapeutics Laboratory, Yale University Schools of Medicine, Engineering and Applied Sciences, 300 George Street, 793 - 748C, New Haven, CT 06511 USA

**Keywords:** Diabetes, Mitochondria, Mitochondrial dynamics, ROS (reactive oxygen species), ROS-induced ROS-release, Ischemia, Ischemia-reperfusion injury, Arrhythmia

## Abstract

Mitochondria play a central role in cellular energy metabolism, and their dysfunction is increasingly recognized as a critical factor in the pathogenesis of diabetes-related cardiac pathophysiology, including vulnerability to ischemic events that culminate in myocardial infarction on the one hand and ventricular arrhythmias on the other. In diabetes, hyperglycemia and altered metabolic substrates lead to excessive production of reactive oxygen species (ROS) by mitochondria, initiating a cascade of oxidative stress that damages mitochondrial DNA, proteins, and lipids. This mitochondrial injury compromises the efficiency of oxidative phosphorylation, leading to impaired ATP production. The resulting energy deficit and oxidative damage contribute to functional abnormalities in cardiac cells, placing the heart at an increased risk of electromechanical dysfunction and irreversible cell death in response to ischemic insults. While cardiac mitochondria are often considered to be relatively autonomous entities in their capacity to produce energy and ROS, their highly dynamic nature within an elaborate network of closely-coupled organelles that occupies 30–40% of the cardiomyocyte volume is fundamental to their ability to exert intricate regulation over global cardiac function. In this article, we review evidence linking the dynamic properties of the mitochondrial network to overall cardiac function and its response to injury. We then highlight select studies linking mitochondrial ultrastructural alterations driven by changes in mitochondrial fission, fusion and mitophagy in promoting cardiac ischemic injury to the diabetic heart.

## Introduction

Diabetes mellitus is a global public health epidemic that continues to expand in both its incidence and prevalence. Among diabetics, cardiac dysfunction, stemming from both ischemic and non-ischemic insults, is the leading cause of morbidity and mortality [1]. Non-ischemic factors comprise diabetic cardiomyopathy, a condition characterized by structural and functional changes in the heart muscle that are independent of pre-existing coronary artery disease. These changes culminate in left ventricular hypertrophy, myocardial fibrosis, and impaired myocardial relaxation, driven largely by a triad of hyperglycemia, insulin resistance, and dyslipidemia. On the other hand, ischemic factors, which this review article focuses on, involve a heightened prevalence and severity of acute ischemia-reperfusion (I/R) events, leading to myocardial infarction, ischemic heart failure and sudden cardiac death due to ventricular arrhythmias.

Central to the development and exacerbation of ischemic insults in the diabetic heart is a state of oxidative stress, which arises from an imbalance in the production and scavenging of reactive oxygen species (ROS), favoring the former. Multiple sources contribute to elevated ROS production in the diabetic heart, including advanced glycation end-products (AGEs) and their receptors (RAGE), NADPH oxidases, uncoupled nitric oxide synthases and most importantly the mitochondrial network. In diabetes, the rate of glucose oxidation is decreased while that of fatty acid oxidation is enhanced, contributing to an increase in oxygen consumption [2]. This fundamental change in substrate utilization in favor of fatty acid oxidation plays a significant role in promoting mitochondrial ROS overproduction. In animal models and human samples from diabetic subjects, mitochondria are prime sources of excess ROS [3, 4]. For example, defective mitochondrial respiration associated with substrates of complexes I, II, and IV, as well as impaired state 4 → 3 transition occurs in isolated mitochondria from cardiomyocytes of leptin receptor deficient mice [5]. This, in turn, causes a major overflow of mitochondrial ROS that is exacerbated by high glucose conditions [5]. While mitochondria are clearly a major source of ROS overproduction in cardiomyocytes, they are also a victim of their own ROS generation. This establishes a vicious mitochondria-centric cycle that culminates in global oxidative stress.

Although mitochondria are often depicted as autonomous organelles that generate ATP and ROS through their intrinsic electron transport chain machinery, they comprise highly dynamic networks whose ultrastructural organization, distribution and morphology significantly impact their function both individually and as a network. The nature of these intricate networks depends on the balance between mitochondrial dynamics proteins, namely those governing biogenesis, fission, fusion, and mitophagy. In this article, we describe: (1) the importance of mitochondrial network dynamics in amplifying ROS injury to the heart culminating in arrhythmias or myocardial infarction, (2) the factors that dictate changes in the mitochondrial network morphology from biogenesis to mitophagy and their alterations in diabetes, and (3) the regulation of these processes by upstream metabolic signaling through the master metabolic sensor, AMPK. Understanding these mechanisms is crucial for developing targeted therapeutic strategies to address cardiovascular complications in diabetic patients.

## Cardiomyocyte mitochondrial network dynamics, ROS-induced ROS-release and global cardiac dysfunction

**Fig. 1:**
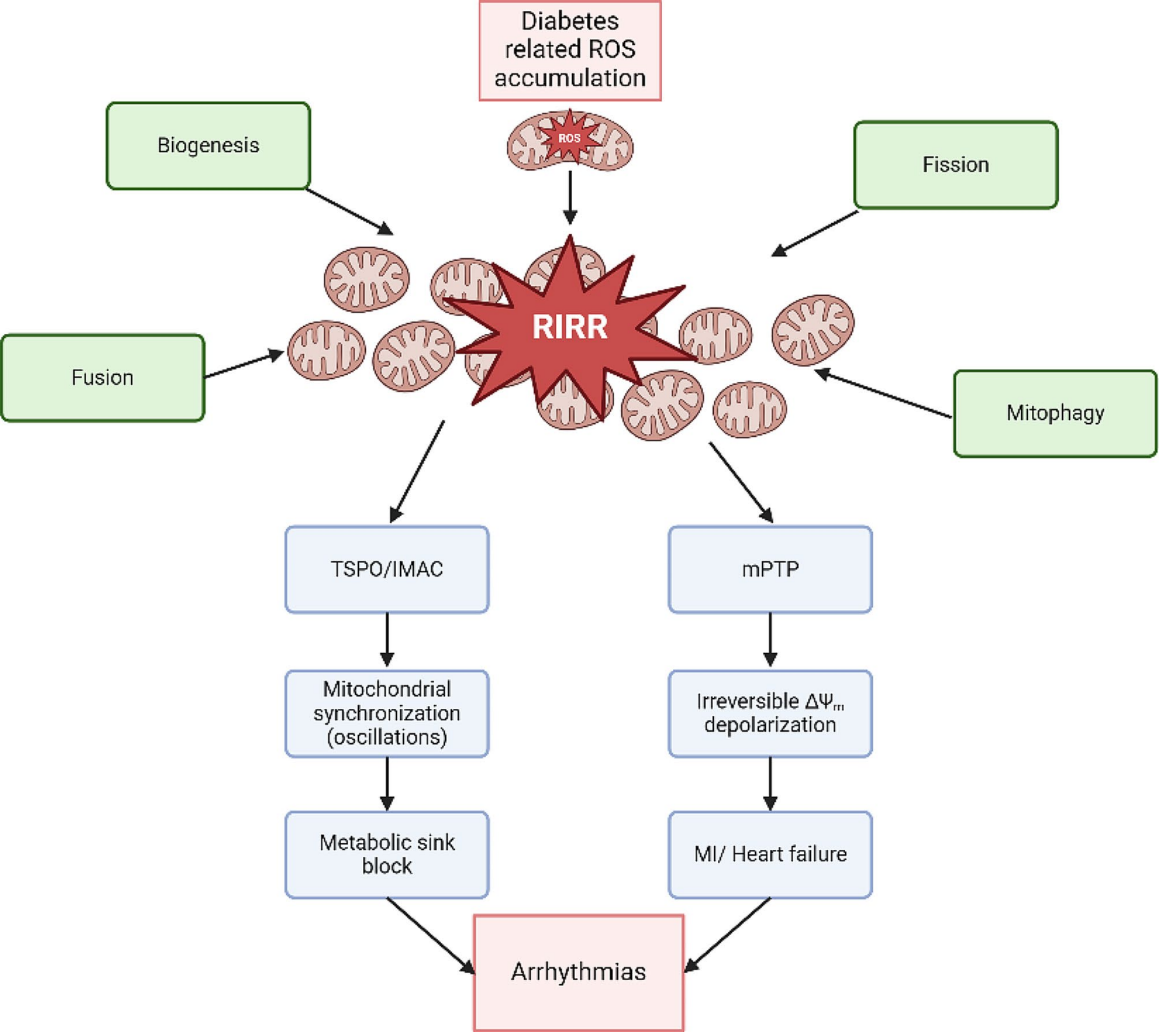
Schematic illustrating the functional and physical features of mitochondrial network dynamics. The processes of biogenesis, fusion, fission and mitophagy dictate the morphology, density and ultrastructure of the mitochondrial network within the cardiomyocyte, which in turn affect the functional coupling/synchronization of individual mitochondria. An increase in mitochondrial production can lead to rapid amplification of ROS across the mitochondrial network via activation of ROS-sensitive mitochondrial channels. A hierarchal activation pattern of ROS sensitive channels causes early activation of TSPO/IMAC leading to reversible mitochondrial membrane potential oscillations that drive electrophysiological instability via cyclical activation of sarcolemmal ATP-sensitive K channels. This promotes conduction abnormalities and arrhythmias. On the other hand, higher levels of oxidative stress can activate the mPTP to cause irreversible mitochondrial depolarization leading to cell necrosis and myocardial infarction, a major risk factor for malignant arrhythmias and sudden cardiac death

Mitochondria are central mediators of the cardiac response to oxidative stress (OS), as they can either amplify or limit ROS-induced injury through a host of ROS-sensitive mitochondrial ion channels [6–8]. The inner membrane anion channel (IMAC) and components of the mitochondrial permeability transition pore (mPTP) are crucial in OS-induced mitochondrial dysfunction. Both channel complexes are activated by increasing ROS levels, but they follow a hierarchical activation pattern [9, 10]. Initially, IMAC responds to moderate OS levels, and subsequently, the large conductance mPTP activates, causing irreversible mitochondrial membrane potential (ΔΨ_m_) depolarization [10]. Indeed, both channels have been implicated in mitochondrial dysfunction through a regenerative, autocatalytic process known as ROS-induced ROS-release (RIRR) which can culminate in electrical dysfunction or cell death (Fig. 1) [11–15].

**Fig. 2:**
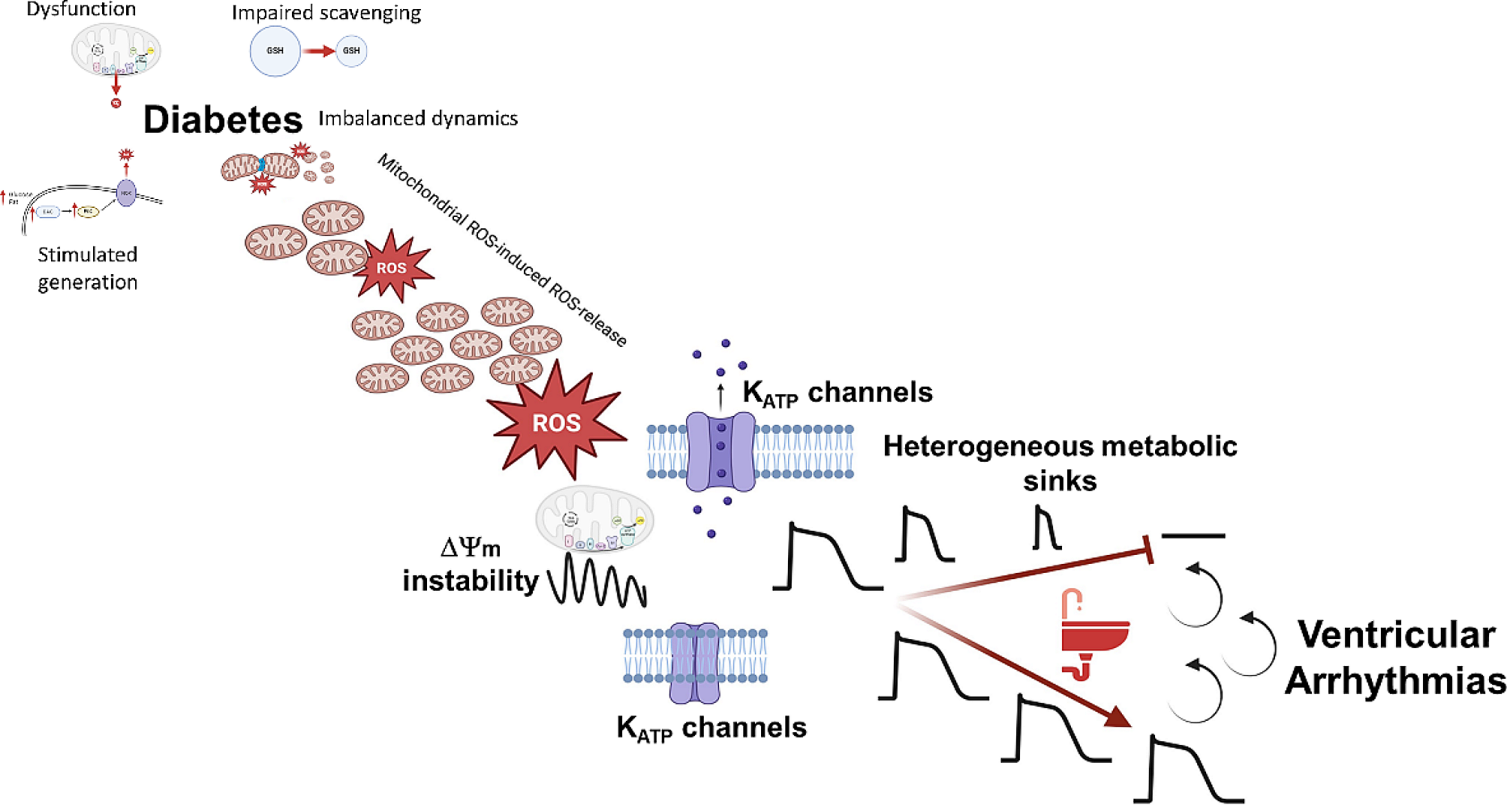
Schematic illustrating the multi-scale process by which individual mitochondria can lead to organ level arrythmias. Diabetes causes an increase in ROS production and impairment in ROS scavenging. ROS levels are amplified through an autocatalytic feed-forward process of ROS-induced ROS-release which culminates in metabolic oscillations and rapid mitochondrial uncoupling. This causes activation of ATP-sensitive potassium channels resulting in membrane inexcitability at the cellular level and a phenomenon termed metabolic sink that leads to conduction block at the tissue level. Heterogeneous formation of these metabolic sinks results in ventricular arrhythmias

RIRR, a fundamental mechanism by which cardiac mitochondria respond to elevated ROS levels by stimulating their own endogenous ROS production, is an emergent property of the mitochondrial network, which depends not only on ROS-sensitive mitochondrial ion channels, but also on the ultrastructure, distribution, morphology and functional coupling of mitochondria within the cardiomyocyte [9, 16]. Once a threshold level of ROS is exceeded across a critical mass of the mitochondrial population within the cardiomyocyte, emergent network behavior in the form of IMAC-mediated synchronized metabolic oscillations or mPTP -mediated global mitochondrial collapse can arise (Fig. 1) [17]. The former involve cell-wide ΔΨ_m_ oscillations that significantly impact cardiomyocyte function and lead to inexcitability at the cellular level through cyclical activation of ATP-sensitive potassium channels [17–19]. At the organ level, heterogeneity in ΔΨ_m_-driven inexcitability promotes a form of conduction failure via a mechanism termed metabolic sink (Fig. 2) [17]. Stabilization of ΔΨ_m_ using pharmacological interventions that target the mitochondrial translocator protein (TSPO) to inhibit IMAC can lead to action potential (AP) stabilization and prevention of post-ischemic arrhythmias [17]. More sustained ROS induced injury, on the other hand, has been shown to activate mPTP culminating in irreversible cellular damage and death leading to cellular necrosis and myocardial infarction, which is a known substrate for malignant ventricular arrhythmias and sudden cardiac death (Fig. 1) [20, 21].

Using various experimental and computational approaches, we and others described the biophysical properties and functional consequences of RIRR at the tissue-network level and across broad myocardial regions. Zhou et al. developed a mathematical, reaction–diffusion model of RIRR that emphasized the importance of superoxide (O_2_^−^) diffusion in mediating mitochondrial dysfunction across 2-dimensional *in silico* networks of virtual mitochondria [22]. We experimentally tested these model predictions using a semi-quantitative approach of O_2_^−^ mapping across the heart [14]. Consistent with cellular studies of RIRR, we demonstrated that exposure of intact hearts to high doses of exogenous pro-oxidants, such as H_2_O_2_, provoked two distinct ROS peaks. While the initial low amplitude peak coincided with the exogenous stressor, the secondary large amplitude peak occurred following, not during the exogenous stress, consistent with a regenerative RIRR response [14]. Functionally, hearts that exhibited the autocatalytic secondary ROS peak were prone to ventricular arrhythmias, whereas those that did not were relatively immune [14]. In a subsequent study, we investigated the relationship between the stability of the mitochondrial membrane in response to oxidative stress and the pro-arrhythmic potential of guinea pig hearts [23]. Specifically, we modulated the threshold and rate of decline of ΔΨ_m_ in response to exogenous pro-oxidant challenge using a variety of agents that affected the activities of key mitochondrial ion channels and their interaction with one another. In doing so, we uncovered functional cross-talk between the energy-dissipative mPTP and the cardioprotective mitochondrial K-ATP channels that ultimately governed the arrhythmic response of the heart to pro-oxidant challenge [23]. Once again, hearts that exhibited rapid ΔΨ_m_ decline were associated with low thresholds for sustained arrhythmias [23]. Consistent with the notion that ΔΨ_m_ stability is protective against electrical dysfunction at the organ level, Alleman et al. [24] demonstrated that exercise-mediated protection against reperfusion arrhythmias was indeed associated with and likely dependent upon proper ΔΨ_m_ polarization [24].

Strong mechanistic links between mitochondrial stability, oxidative stress and arrhythmias in the diabetic heart have been documented by multiple groups [16, 25–31]. For example, we found that glutathione (GSH) oxidation and therefore impaired ROS scavenging, were effective in unmasking mitochondrial depolarization and arrhythmic vulnerability of chronically hyperglycemic guinea pigs [32]. Slodzinski et al. [33] used two-photon microscopy to demonstrate that acute oxidative stress by GSH oxidation readily produces heterogeneous fluctuations in ΔΨ_m_ and ROS levels between neighboring myocytes within the intact heart [33]. Since ΔΨ_m_ oscillations can drive action potential duration oscillations [17], spatiotemporal changes in ΔΨ_m_ may indeed increase electrical heterogeneity across the heart, which we found was directly linked to pro-arrhythmic vulnerability in diabetic guinea pigs [32]. Finally, using wavelet analysis of the mitochondrial network’s response to pro-oxidant challenge, Vetter et al [26]. demonstrated that cardiomyocytes from diabetic guinea pig hearts were in a state of heightened vulnerability to cell-wide mitochondrial oscillations compared to their non-diabetic counterparts, an effect that could be rescued by insulin treatment [26]. Collectively, these studies and others emphasized the importance of the functional coupling between individual mitochondria within the cardiomyocyte in the response to injury. Such functional coupling is modulated by the ultrastructural features of the mitochondrial network which is composed of organelles that continuously fuse, divide and turnover in an effort to meet the *changing* energetic requirements of the cardiomyocyte and the organ as a whole [34, 35]. Indeed, remodeling of the mitochondrial network in acquired diseases such as heart failure in favor of fragmented mitochondria have been shown to inhibit the synchronization of metabolic oscillations while sensitizing individual mitochondria to permanent depolarization [36]. While not explicitly investigated, these effects would be expected to suppress post-ischemic arrhythmias at the expense of expanding the infarct size. As will be discussed next, the dynamic morphological adaptations of the mitochondrial network that regulate its function as a unit are orchestrated by fundamental cellular processes, namely mitochondrial biogenesis, fusion, fission, and mitophagy. Furthermore, these processes are remodeled in the context of common metabolic diseases and contribute to the enhanced propensity of the diabetic heart to pro-oxidant injury and post-ischemic arrhythmias, as we and others have observed [5, 25, 32].

## Mitochondrial dynamics and the response of the mitochondrial Network to oxidative stress: from biogenesis to mitophagy

As mentioned previously, a host of ROS sensitive mitochondrial ion channel complexes in the cardiomyocyte activate in a hierarchal manner to produce metabolic oscillations that provoke arrhythmias on the one hand or irreversible mitochondrial depolarization leading to necrosis on the other. The amplification of ROS injury, however, is dependent not only on the intrinsic mitochondrial ion channels within individual mitochondria, but also on their network properties, namely their ultrastructural organization and morphology (Fig. 1). Indeed, cardiac mitochondria are densely packed within a 3-dimensional lattice structure adjacent to the myofilaments and T-tubules of the cardiomyocyte. Their unique network architecture and extensive subcellular organization localizes mitochondria in close spatial proximity to critical loci where: (1) energy is consumed (i.e. the myofilaments), (2) excitation-contraction coupling is initiated (i.e. the dyads), and (3) energy-dependent ion channels, exchangers and transporters are concentrated (i.e. the sarcolemma). While this network organization optimizes the efficient delivery of energy to critical microdomains within the cardiomyocyte, this same proximity also contributes to the transmission and amplification of pro-oxidant injury resulting in global cellular dysfunction and death. In what follows, we briefly introduce the mitochondrial dynamics processes of biogenesis, fission, fusion, and mitophagy, which govern the ultrastructural morphology and architecture of the mitochondrial network. For comprehensive reviews on the subject, the reader is referred to excellent reviews [37, 38].

### Biogenesis

Mitochondrial biogenesis is the orchestrated process by which new mitochondria are generated to maintain or expand the existing mitochondrial pool in a given cell. Under conditions of energy supply-to-demand mismatch, mitochondrial biogenesis acts as a compensatory mechanism to restore mitochondrial capacity of the diabetic heart. Mitochondrial biogenesis requires the activation of various signaling pathways, including peroxisome proliferator-activated receptor gamma coactivator-1 alpha (PGC-1α), nuclear respiratory factors [39], and mitochondrial transcription factor A [40]. The coordinated activation of these factors causes a surge in mitochondrial mass, which bolsters the respiratory capacity and bioenergetic status of the cardiomyocyte; thereby, enhancing cardiac function in the face of increased metabolic demands.

### Fission and fusion

In the native myocardium, fission and fusion events are required to maintain mitochondrial integrity and metabolic homeostasis [41]. Disruption of these opposing processes represents an early response to stress and has been observed in the onset and progression of cardiac disorders [41]. Mitochondrial fusion and fission are mediated by mechanisms that determine both the ultrastructure and function of mitochondria. Fusion describes the merger of the outer and inner membranes (OMM and IMM, respectively) of adjacent mitochondria, leading to the formation of elongated and interconnected networks with synchronized DY_m_, a key functional metric that fuels electron transport across the inner membrane. In addition to promoting functional coupling across the mitochondrial network, fusion also plays a crucial role in maintaining uniform mitochondrial DNA within neighboring mitochondria [42]. The process of mitochondrial fusion is coordinated by specific membrane-bound GTPases, namely mitofusins 1 & 2 (Mfn1 & Mfn2) and optic atrophy protein 1 (Opa1) [43–46]. Mitofusins form tethering connections which undergo GTP hydrolysis-driven conformational changes that merge the OMMs of neighboring mitochondria. A similar process involving Opa1 and cardiolipin mediates the fusion of the IMM; thereby completing the union of the two adjacent organelles [47].

Mitochondrial fission, on the other hand, is an evolutionarily conserved process that facilitates the division of mitochondria. Mitochondrial fission serves multiple purposes that are essential to cellular life, including the regulation of mitochondrial inheritance, the removal of damaged organelles, and the release of pro-apoptotic factors [42]. The primary mediator of mitochondrial fission is the dynamin-related protein 1 (Drp1), also known as dynamin-1-like protein. Drp1 is recruited from the cytoplasm to specific sites on the OMM [48, 49] through interactions with various Drp1 adaptor proteins, including mitochondrial fission 1 (Fis1), mitochondrial fission factor (Mff) and others [50–52]. Binding of Drp1 by these adaptors leads to the formation of Drp1 multimeric spirals that trigger GTP hydrolysis to induce a constricting conformational change [53]. This, in turn, facilitates the severing of the inner and outer mitochondrial membranes of the dividing organelles.

Changes in Drp1 expression alone are often insufficient to alter mitochondrial fission; instead, this process is highly regulated by post-translational modifications and their interactions with various OMM sites. These modifications can vary under different pathophysiological conditions. For example, the phosphorylation of Ser-600 and Ser-637 have been shown to be particularly important in diabetic mouse models [54–57].

### Mitophagy

As mentioned above, mitochondrial fission is a critical process that serves to segregate damaged mitochondrial components thereby enhancing their accessibility for selective clearance, which then occurs through the process of mitophagy. Mitophagy, a specialized form of autophagy, recognizes damaged mitochondria, marking them for subsequent degradation. Hence, the cooperative interplay between fission and mitophagy serves as a pivotal mechanism for safeguarding the structural and functional integrity of the mitochondrial network. Alterations in mitophagy rates, either through unregulated activation or impaired function, contribute to the progression of many cardiovascular diseases [58]. At the molecular level, PTEN-induced kinase 1 (PINK1), a mitochondrial serine/threonine-protein kinase converges on damaged mitochondria, leading to the recruitment and subsequent ubiquitination of mitochondrial OMM proteins by Parkin. This, in turn, facilitates the binding of autophagy receptors.

The impact of altered mitochondrial dynamics on cardiac dysfunction specifically in the context of diabetes mellitus will be explored in more detail next [59]. We will focus on recent advances in our understanding of how alterations in mitochondrial network ultrastructure and morphology, driven by changes in mitochondrial dynamics and mitophagy-related proteins, impact myocardial function of the diabetic heart as well as its response to ischemic injury.

## Mitochondrial network dysfunction in the diabetic heart

A frequently observed characteristic in tissues of hyperglycemic patients and animal models is mitochondrial deformation, marked by excessive accumulation of fragmented mitochondria [60, 61]. The importance of these ultrastructural changes is underscored by the fact that mitochondrial homeostasis and function are reflected by their morphology. Perhaps more importantly, changes in mitochondrial morphology have often been shown to cause, rather than merely reflect the increased generation of ROS in hyperglycemic conditions. Specifically, Yu et al. [60] elegantly demonstrated that mitochondrial fragmentation mediated by the fission process is a necessary component of high glucose-induced ROS overproduction and altered mitochondrial respiration in H9c2 cells. Overexpression of a dominant negative form of Drp1 prevented high glucose-induced mitochondrial fragmentation and excessive ROS production in cell lines. In another study, Drp1 silencing using small interfering RNAs in H9c2 cells exposed to oxidative stress also resulted in reduced mitochondrial fragmentation and improved insulin signaling [62]. Similarly, overexpression of Mfn2 or Opa1 were both effective in improving mitochondrial function and suppressing ROS in response of various cells to high glucose exposure [63, 64]. These lines of evidence strongly suggest that mitochondrial dynamics may be a previously unrecognized nexus in the control of ROS production in hyperglycemia-associated disorders [63].

The functional significance of mitochondrial fission during myocardial I/R injury is evident from numerous studies demonstrating a potent cardioprotective efficacy of Drp1 inhibition or gene silencing [65–69]. For example, in HL-1 cells, expression of a dominant negative Drp1 mutant resulted in the genesis of elongated mitochondria with reduced sensitivity to mitochondrial permeability transition [65]. Accordingly, transgenic expression of this mutant in rat hearts resulted in decreased infarct size, reduced cell death, and improved cardiac function following in vivo I/R [68]. Moreover, indirect suppression of Drp1 through the kinase Pim-1, known for its antiapoptotic and pro-proliferative properties, preserved mitochondrial phenotype in neonatal rat cardiomyocytes exposed to simulated ischemia [67]. Finally, pharmacological Drp1 inhibition using Drpitor1a preserved cardiac function during I/R, likely by inhibiting mitochondrial fission [66]. Taken together, these studies and others demonstrate the cardioprotective effect of preventing mitochondrial fission through pharmacological, regulatory and gene-based Drp1 downregulation during I/R injury. The role of Drp1 in the development of I/R injury specifically in the context of diabetes, has received limited attention thus far [60, 62, 70, 71]. A recent study in a mouse model of diabetes provided compelling evidence that increased translocation of Drp1 to mitochondria during I/R injury was associated with decreased mitochondrial size, consistent with a pro-fission response [72]. In vivo administration of MDIVI-1 to these mice inhibited Drp1 translocation to mitochondria, reduced mitochondrial fission, limited the extent of myocardial infarction and reduced levels of serum cardiac troponin and lactate dehydrogenase [72]. Similarly, in a rat model of high fat diet, treatment with MDIVI1 before, during or even after the ischemic challenge were effective in decreasing mitochondrial ROS generation, ΔΨ_m_ depolarization and arrhythmia burden [73]. Altering mitochondrial dynamics by promoting fusion via the small molecule M1 (which targets Opa1) was found to be equally effective in mitigating the detrimental effects of I/R injury in prediabetic animals as that achieved using the chemical Drp1 inhibitor MDIVI1 [74]. Interestingly, the combined treatment with both agents did not appear to exert added benefit over mono-therapy by MDIVI1 or M1 alone [75].

It is important to note, however, that proteins that regulate mitochondrial dynamics also serve important functions that are independent of fusion or fission, per se. For example, Mfn2 plays a key role in mediating functional coupling between mitochondria and the sarco(endo)plasmic reticulum (SR/ER) via physical tethering of the two organelles [76]. Hyperglycemia increases mitochondria–ER contact, alters Ca^2+^ levels within both compartments to promote mitochondrial ROS production, ER stress, mitochondrial dysfunction, and apoptosis. These effects of hyperglycemia are reversed by Mfn2 gene silencing, which was reported to decrease Ca^2+^ transfer from the ER to mitochondria; thereby ameliorating mitochondrial ROS production, oxygen consumption, mitochondrial dysfunction and cell death [77, 78]. In contrast, exaggerated mitochondrial Ca^2+^ uptake in models of prediabetes due to increased mitochondria-SR tethering can exacerbate adverse remodeling and increase mortality [79, 80].

Finally, mitophagy plays a major role in the regulation of cardiac function in diabetes by limiting the extent of oxidative stress through elimination of dysfunctional mitochondria [81]. Mitophagy, which is typically stimulated under conditions of nutrient deficiency, earmarks damaged mitochondria for lysosome-mediated degradation [82]. Indeed, increased mitophagy has been identified as a relatively early event that precedes overall cardiac dysfunction and likely plays a cardioprotective role in models of pre-diabetes. To that end, injection of Beclin-1 conjugated to a Tat peptide was found to be effective in ameliorating mitochondrial dysfunction, decreasing lipid accumulation, and protecting against diastolic dysfunction in mice on a high fat diet due to activation of mitophagy [83]. Finally, in chronic high fat diet models conventional mitophagy is inactivated, while an alternative form mediated by Ulk1 and Rab9-dependent processes is activated [84–87]. Table 1 summarizes some of the key studies linking changes in mitochondrial dynamics protein expression to network ultrastructure and cardiac function in diabetes that we covered in this section.

**Table 1:**
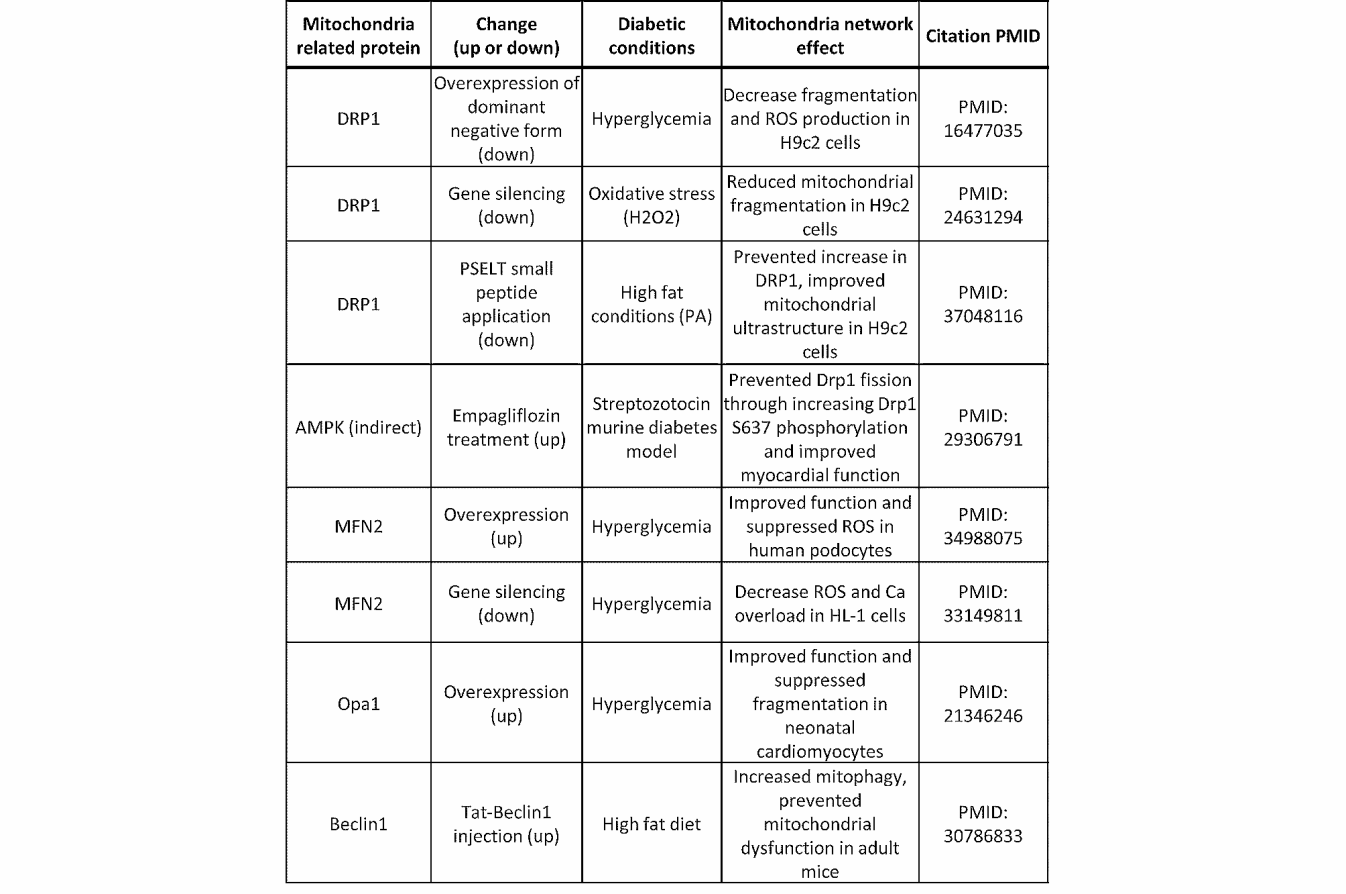
Select studies linking changes in mitochondrial dynamics protein expression to network ultrastructure and cardiac function in diabetes

## Regulation of mitochondrial dynamics by upstream metabolic signaling: role of AMPK

Upstream metabolic signaling via the master metabolic sensor 5’ adenosine monophosphate-activated protein kinase (AMPK) is vital in the diabetic heart, particularly in its response to ischemic injury. Diabetes exacerbates cardiovascular complications by impairing metabolic flexibility and energy homeostasis, placing the heart at a heightened risk to ischemic injury. AMPK functions as a cellular energy sensor, activated by an increase in the AMP/ATP ratio, which is a common occurrence during ischemic stress. In addition, AMPK is activated by the upstream kinases LKB1 and CaMKKβ (Ca^2+^/calmodulin-dependent protein kinase β). LKBI activation of AMPK is intertwined with AMP binding to the AMPKγ-subunit. In contrast, CaMKKβ activates AMPK in response to increases in cellular Ca^2+^ without significant changes in AMP/ADP/ATP levels [88, 89]. The activation of AMPK by these various substrates was originally thought to be tissue specific, with the primary activators in the heart being AMP, ADP, and LKB1. More recent data have revealed the importance of CaMKK2 in the reduction of oxidative stress and inflammation via the AMPK-AKT-GSK-3beta/Nrf2 pathway in response to myocardial I/R injury [90].

In addition to cellular stressors such as low nutrients or prolonged exercise, AMPK is also activated by various pharmacologic agents (see full review Mihaylova & Shaw, 2011) [89]. Most relevant to this review is the activation of AMPK by metformin, the most widely prescribed Type 2 diabetes drug. Metformin activates AMPK in a LKB1 dependent manner. Indeed, Shaw et al. have shown that deletion of LKB1 in the adult mouse liver leads to nearly complete loss of AMPK activity and that metformin requires hepatic LKB1 to lower blood glucose levels [91]. Metformin, however, is an AMPK agonist that also blocks mitochondrial complex I in many cell types and in doing so elicits numerous pleiotropic effects that impact cardiac function. These include a reducion of glucose output from the liver, a decrease in glycation end products and ROS production in the endothelium, and altered regulation of glucose and lipid metabolism in cardiomyocytes [92].

Upon activation, AMPK restores energy balance by stimulating catabolic processes such as glucose uptake, enhancing glycolysis, and promoting fatty acid oxidation. Additionally, AMPK activation initiates protective autophagy and mitophagy processes that remove damaged cellular components and dysfunctional mitochondria, thus preserving cellular integrity. By regulating these adaptive responses, AMPK not only mitigates the detrimental effects of ischemic injury in the diabetic heart but also improves overall cardiac function and survival. In what follows, we highlight the intricate control of mitochondrial biogenesis, fission, fusion and mitophagy by AMPK signaling in the context of diabetes.

### AMPK regulation of mitochondrial biogenesis

AMPK plays a critical role in the regulation of mitochondrial biogenesis in skeletal muscle [39, 93, 94] and likely in heart [95, 96]. Upon activation, AMPK enhances the activity of PGC-1α (peroxisome proliferator-activated receptor gamma coactivator 1-alpha), a key transcriptional coactivator that drives the expression of genes involved in mitochondrial biogenesis [97]. In turn, PGC-1α activates several nuclear transcription factors, including NRF-1 (nuclear respiratory factor 1) and NRF-2, which promote the expression of Tfam (mitochondrial transcription factor A, also known as mtTFA), leading to the transcription of mitochondrial DNA-encoded and nuclear-encoded mitochondrial genes [39, 40, 98]. In addition to transcriptional activation through NRFs, PGC-1α is also the co-activator of the PPARs family, thyroid hormone, glucocorticoid, oestrogen, and ERRs (oestrogen-related receptors) [40, 99]. Thus, the activation of PGC-1α via AMPK leads to the regulation of energy homeostasis via the synthesis of new mitochondrial proteins and the formation of new mitochondria, thereby enhancing the oxidative capacity of cardiac cells.

In addition to its role in activating PGC-1α, AMPK influences mitochondrial biogenesis through other signaling pathways. Whereas PGC-1α has been found to activate mitochondrial biogenesis in response to low temperature and prolonged exercise, activation of SIRT1 (sirtuin 1) occurs in response to fasting through a nutrient-signaling response [40, 97, 100, 101]. Indeed, AMPK directly phosphorylates and activates SIRT1, a deacetylase that further activates PGC-1α [100]. This, in turn, amplifies the mitochondrial biogenesis response. Finally, AMPK regulates the expression of mitochondrial genes by directly phosphorylating and activating transcription factors that are crucial for mitochondrial DNA replication and transcription [39, 40, 98].

The regulation of mitochondrial biogenesis by AMPK is particularly important in the context of cardiac diseases, such as heart failure and ischemic heart disease, where mitochondrial dysfunction is a key pathological feature and energy demand often exceeds supply. In this context, AMPK activation can support cellular survival by expanding the mitochondrial pool to increase ATP synthesis. Moreover, AMPK deficiency has been reported to result in a significant decrease in PGC-1α driven mitochondrial biogenesis in aged hearts that are prone to ROS production and concomitant contractile dysfunction [102]. Both genetic knockout of PGC-1α and pharmacologic competition for its coactivator, PPARα/γ, lead to inhibition of the PGC-1α/SIRT1 pathway, lower mitochondrial abundance, and decreased cardiac function [95, 103]. Therefore, targeting AMPK signaling to enhance mitochondrial biogenesis represents a promising therapeutic strategy to improve cardiac function and resilience in various heart diseases and conditions, such as aging [41].

### AMPK regulation of mitochondrial fission and fusion

Multiple lines of evidence have highlighted the robust regulation of mitochondrial fission in the heart by AMPK signaling [70, 104–106]. AMPK has multiple phosphorylation targets leading to mitochondrial fission and fusion, including Mff, ARMC10 (Armadillo repeat-containing protein 10 ), and MTFR1L (mitochondrial fission regulator 1-like protein). Pharmacological activation of AMPK directly promotes mitochondrial fission by phosphorylating serine-155 and serine-173 on Mff [107], which is involved in the recruitment of Drp1 to the mitochondrial membrane [108]. In fact, Drp1 localizes to mitochondria only when the AMPK phosphorylation sites on Mff are intact. Another key substrate of AMPK phosphorylation is the serine-45 site on ARMC10 [109]. ARMC10 localizes to the OMM and interacts with Mff and Drp1 to modulate mitochondrial fission. Lastly, AMPK has been shown to phosphorylate MTFR1L, and in doing so, to promote mitochondrial fragmentation in response to stress [110]. In this regard, depletion of AMPK was found to abolish mitochondrial fission induced by inhibitors of mitochondrial complexes I or III [106]. Yet, the regulation of mitochondrial fission by AMPK is multi-factorial and complex as a growing body of evidence has shown that increases in AMPK signaling inhibit pathological fission through direct phosphorylation of Drp1 at Ser-637, and in doing so to protect against cardiac dysfunction in a variety of settings.

As mentioned previously, AMPK mediates mitochondrial fission through direct targeting of Mff and MTFR1L. In the context of diabetes, however, activation of AMPK-mediated cardioprotective signaling has been shown to inhibit rather than promote pathological mitochondrial fission by altering Drp1 phosphorylation at Ser-637 [70]. In fact, there is a growing body of evidence suggesting the crucial involvement of the so-called AMPK-Drp1 axis in the cardioprotective effects of various interventions in type 2 diabetes mellitus. For instance, Zhou et al. [70] demonstrated that the sodium/glucose cotransporter 2 inhibitor Empagliflozin improved myocardial function, preserved microvascular barrier integrity, sustained endothelial nitric oxide synthase phosphorylation, and enhanced endothelium-dependent relaxation through AMPK-dependent inhibition of mitochondrial fission [70]. Together these data show the importance of AMPK in the regulation of mitochondrial dynamics and function in the diabetic heart.

### AMPK regulation of mitophagy

As with mitochondrial biogenesis and dynamics, AMPK is also a major regulator of mitophagy. AMPK activates Unc-51-like Kinase 1 (ULK1), a key initiator of mitophagy, directly by phosphorylation as well as indirectly through suppression of mTOR signaling. AMPK-dependent activation of ULK1 promotes autophagosome formation around dysfunctional mitochondria and has also been suggested to activate Pink1/Parkin dependent mitophagy [111, 112]. Of note, an alternative mitophagy process mediated by a Parkin-independent, but Ulk1-Rab9 dependent, mechanism has also been documented in various settings by the Sadoshima group [84–86]. Finally, mitochondria-localized pools of AMPK (so-called mitoAMPK) are activated by local energetic stress to induce mitophagy, at least in skeletal muscle; thereby, acting as local sensors that help maintain quality control [113]. AMPK is the functional connection between energy sensing and mitochondrial homeostasis given its role in the biogenesis of new mitochondria via activation of PGC-1α and its regulation of mitophagy through a ULK1-dependent mechanism.

Additionally, AMPK enhances mitophagy at the transcriptional level by regulating the expression of genes involved in autophagic and lysosomal pathways. It upregulates proteins such as BNIP3 (BCL2/adenovirus E1B 19 kDa interacting protein 3) and NIX (NIP3-like protein X), which are essential for the recognition and targeting of damaged mitochondria for autophagic degradation. Furthermore, AMPK influences the activity of TFEB (transcription factor EB), a master regulator of lysosomal biogenesis and function. By promoting the nuclear translocation and activity of TFEB, AMPK ensures an adequate supply of lysosomal enzymes and an efficient degradation capacity, facilitating the clearance of damaged mitochondria.

Finally, AMPK also exerts strong regulation over mitochondrial function independently of its influence on network dynamics. Indeed, cardiac mitochondria isolated from mice expressing a kinase dead mutant of AMPK exhibit reduced oxidative capacity, increased H_2_O_2_ production and decreased resistance to mitochondrial permeability transition pore opening [114]. These intrinsic mitochondrial deficits correlate with increased rates of necrosis during reperfusion after coronary occlusion [114]. In this context, it is important to note that AMPK signaling in diabetes appears to be salutary in nature both in terms of its effects on individual mitochondria and the network as a whole.

### Key unresolved questions

Despite the robust body of work linking mitochondrial dynamics to the cardiovascular pathophysiology of I/R injury, it is worth noting that contradictory evidence does exist in the literature that suggests a more nuanced role of mitochondrial fission and fusion in the regulation of I/R injury as highlighted in Table 2. For example, cardiac-specific Drp1 knockout mice exhibit greater mitochondrial dysfunction, left ventricular remodeling, and premature death than their control counterparts [115]. This strongly suggests that a basal level of myocardial Drp1 expression is likely required for maintenance of physiological mitochondrial function. Mfn2 deletion, which increases the rate of mitochondrial fission, was also reported in one study to paradoxically prevent rather than promote cell death and to reduce rather than expand the size of the infarct [116]. Silencing of Mfn2 in H9c2 cells or Mfn1 in cardiomyocytes also elicited an unexpected protection against ROS-induced apoptosis [117, 118]. Furthermore, overexpression of Opa1 did not confer protection against apoptosis induced by simulated ischemia in H9c2 cells [119]. These seemingly paradoxical findings emphasize the need for further examination to fully understand the precise role of mitochondrial dynamics in the context of I/R injury in various settings. These studies also indicate that therapeutic treatments to manipulate mitochondrial dynamics processes might need to be restricted to acute interventions because chronic alterations in the balance between fusion and fission can potentially disrupt adaptive mechanisms that are required for long-term mitochondrial and cardiac homeostasis. It is also likely that achieving cardioprotection against I/R might require a more nuanced orchestrated activation of multiple mitochondrial dynamics proteins, rather than more dramatic manipulation of single targets that may disrupt mitochondrial feedback processes. In this regard, AMPK activation through its salutary regulation of mitochondrial network dynamics from biogenesis to mitophagy represents an attractive target. Nonetheless, acute pharmacological inhibition or transient gene silencing of Drp1 at the time of injury represent therapeutic strategies that warrant further development and testing. Indeed, the mitochondrial division inhibitors MDIVI-1, Drpitor1a and P110, which act on Drp1 through distinct mechanisms, have all shown promising effects in reducing myocyte apoptosis in the context of ischemic injury, both in vitro and in vivo [120]. Moreover, customizing the therapeutic approach to specific molecular targets (Mfn2, Opa1, Drp1 or others) is likely to be required for achieving an optimal therapeutic effect. In addition, therapeutic strategies will also have to avoid potentially deleterious non-canonical processes, such as interfering with mitophagy in the case with Drp1 [121] or mitochondria/SR tethering in the case with Mfn2 [122, 123].

**Table 2:**
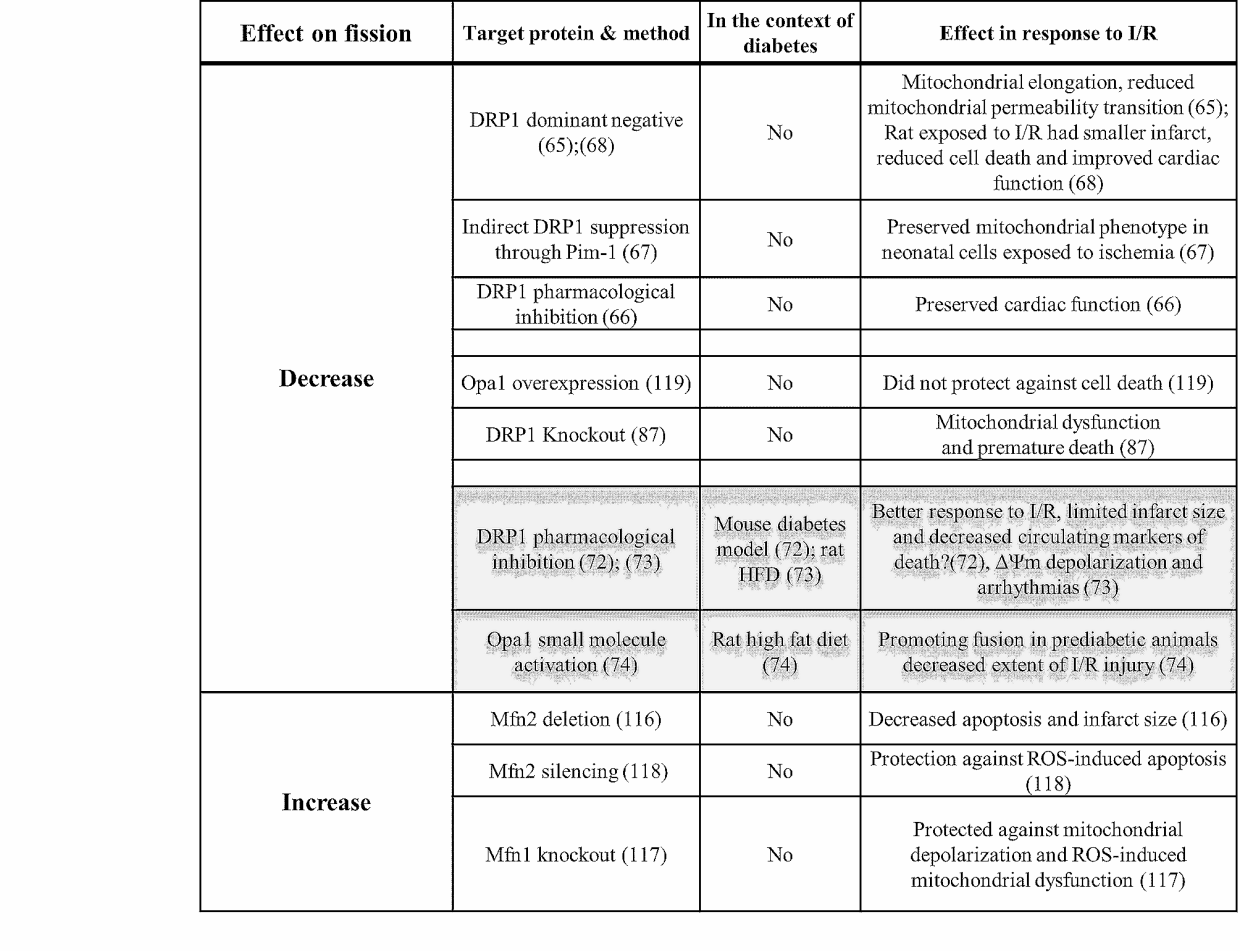
Select studies showing inconsistent effects of altering mitochondrial fission/fusion on I/R injury

## Conclusions

Diabetes is associated with a very high burden of coronary artery disease leading to myocardial infarction (MI) and ventricular arrhythmias [124]. Central to the pathophysiology of the diabetic heart and its susceptibility to acute and chronic ischemic injuries is the mitochondrial network which amplifies ROS injury to produce metabolic oscillations underlying arrhythmias or irreversible depolarization leading to cell death. The delicate interplay of mitochondrial biogenesis, fusion, fission and mitophagy is crucial for adapting to the physiological and pathological demands of the cardiomyocyte as a whole. Ongoing investigations in our laboratories are focused on understanding the regulation of cardiac function and arrhythmogenesis by upstream metabolic signaling that controls mitochondrial biogenesis, dynamics and mitophagy. It is our hope that these studies will uncover new therapeutic approaches for treating cardiac dysfunction in the context of metabolic diseases by improving mitochondrial function.

## Data Availability

No datasets were generated or analysed during the current study.

## Abbreviations

AGE: Advanced glycation end products
AMPK: 5' adenosine monophosphate-activated protein kinase
AP: Action potential
ARMC10: Armadillo repeat-containing protein 10
BNIP3: BCL2/adenovirus E1B 19 kDa protein-interacting protein 3
CaMKKβ: Ca^2+^/calmodulin–dependent protein kinase β
Drp1: Dynamin-related protein 1
eNOS: Endothelial nitric oxide synthase
ETC: Electron transport chain
Fis1: Mitochondrial fission 1
GSH: Glutathione
IMM: Inner mitochondrial membrane
I/R: Ischemia-reperfusion
JNK: c-Jun N-terminal kinas
Mdivi-1: Mitochondrial division inhibitor 1
mDNA: Mitochondrial DNA
Mff: Mitochondrial fission factor
Mfn: Mitofusin
mPTP: Mitochondrial permeability transition pore
MTFR1L: Mitochondrial fission regulator 1-like protein
nDNA: Nuclear DNA
NRF: Nuclear respiratory factors
OMM: Outer mitochondrial membrane
Opa1: Optic atrophy protein 1
OS: Oxidative stress
PGC-1α: Peroxisome proliferator-activated receptor gamma coactivator 1-alpha
PINK1: PTEN-induced kinase 1
RIRR: ROS-induced ROS-release
ROS: Reactive oxygen species
Sirt: Sirtuin
TFAM: Mitochondrial transcription factor A
TSPO: Mitochondrial translocator protein
UCP: Mitochondrial uncoupling proteins
ULK1: Unc-51-like kinase 1
ΔΨm: Mitochondrial membrane potential
